# Case Report: Regaining radioiodine uptake following PRRT in radioiodine-refractory thyroid cancer: A new re-differentiation strategy?

**DOI:** 10.3389/fnume.2022.1071022

**Published:** 2023-01-17

**Authors:** Bentolhoda Hadad, Emran Askari, Seyed Rasoul Zakavi, Kamran Aryana, Soheila Erfani, Pegah Sahafi, Nima Nabavi, Atena Aghaee

**Affiliations:** Nuclear Medicine Research Center, Mashhad University of Medical Sciences (MUMS), Mashhad, Iran

**Keywords:** RAI-refractory, thyroid cancer, PRRT, somatostatin receptor, lu177-dotatate

## Abstract

A 61-year-old woman with a history of metastatic follicular thyroid carcinoma became radioiodine-refractory following two doses of radioiodine (RAI) therapy (cumulative = 230 mCi). While no RAI-avid lesion was noticed in the last post-ablation whole-body radioiodine scan (WBIS), she reported sternal pain, which was accompanied by rapidly rising thyroglobulin levels. ^18^F-FDG and ^68^Ga-DOTA-TATE PET/CT was performed, showing metastatic pulmonary nodules and a lytic sternal lesion with acceptable avidity (i.e. uptake ≥ liver). Following four cycles of peptide receptor radionuclide therapy (PRRT) with ^177^Lu-DOTA-TATE, the thyroglobulin levels dropped significantly, and the sternal pain was partially alleviated. Despite only experiencing grade I thrombocytopenia, the treating physician decided to discontinue PRRT and repeat the diagnostic WBIS. Surprisingly, the scan revealed significantly increased tracer uptake in the sternum. The patient received 200 mCi ^131^I, and WBIS showed increased RAI uptake in all pulmonary nodules as well as bone metastases. We report a case of RAI-refractory thyroid carcinoma with a somatostatin-receptor expression that re-differentiated and gained significant RAI uptake capacity after PRRT.

## Introduction

The most common endocrine malignancy, differentiated thyroid cancer (DTC), is usually associated with a good prognosis. However, about 10% of DTCs are metastatic, of which two-thirds lose their radioiodine (RAI) uptake and finally become RAI-refractory (RAIR-DTC), heralding a worse prognosis (1, 2). Multiple re-differentiation strategies have been proposed, including tyrosine kinase inhibitors (TKI) and retinoic acid administration (3).

A proportion of RAIR-DTCs also fall into the thyroglobulin (Tg)-elevated negative iodine scan (TENIS) category. These patients are candidates for a variety of imaging modalities (4). While FDG PET/CT is considered the primary imaging modality option in TENIS syndrome patients, imaging with somatostatin analogues (SSTR) is also noteworthy since it provides a chance for peptide receptor radionuclide therapy (PRRT) (4). Although the experience of PRRT in differentiated thyroid cancer is still in its infancy, studies have shown disease control rates similar to TKIs (5, 6). We report a case of TENIS syndrome regaining its RAI uptake following PRRT.

## Case report

**Figure 1:**
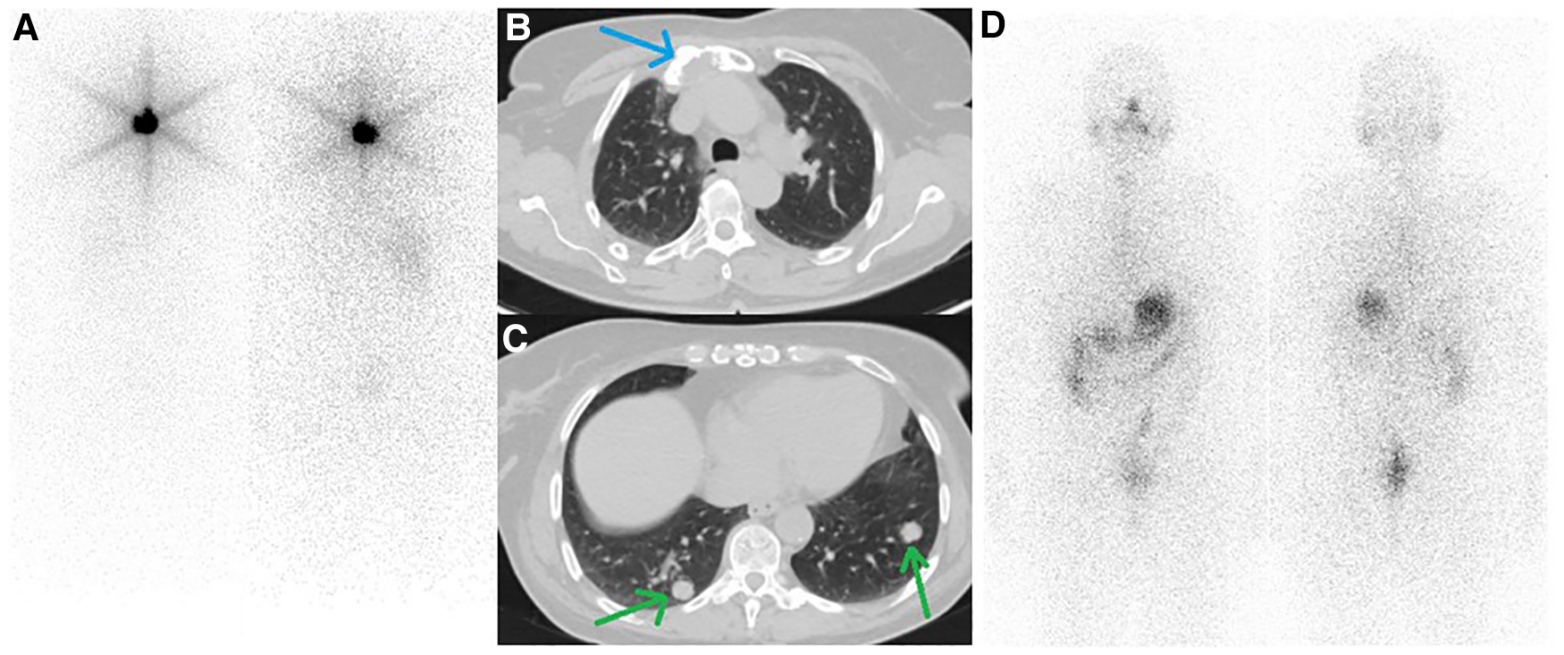
(**A**) First whole-body iodine scan after administration of 30 mCi of ^131^I showed post-surgical thyroid remnant. (**B**, **C**) follow-up CT scan from the thoracic region showed a lytic lesion in the sternum (blue arrow) along with multiple bilateral pulmonary nodules (green arrows). (**D**) The second post-treatment whole-body iodine scan after administration of 200 mCi ^131^I revealed no radioiodine-avidity in the tumoral lesion.

**Figure 2:**
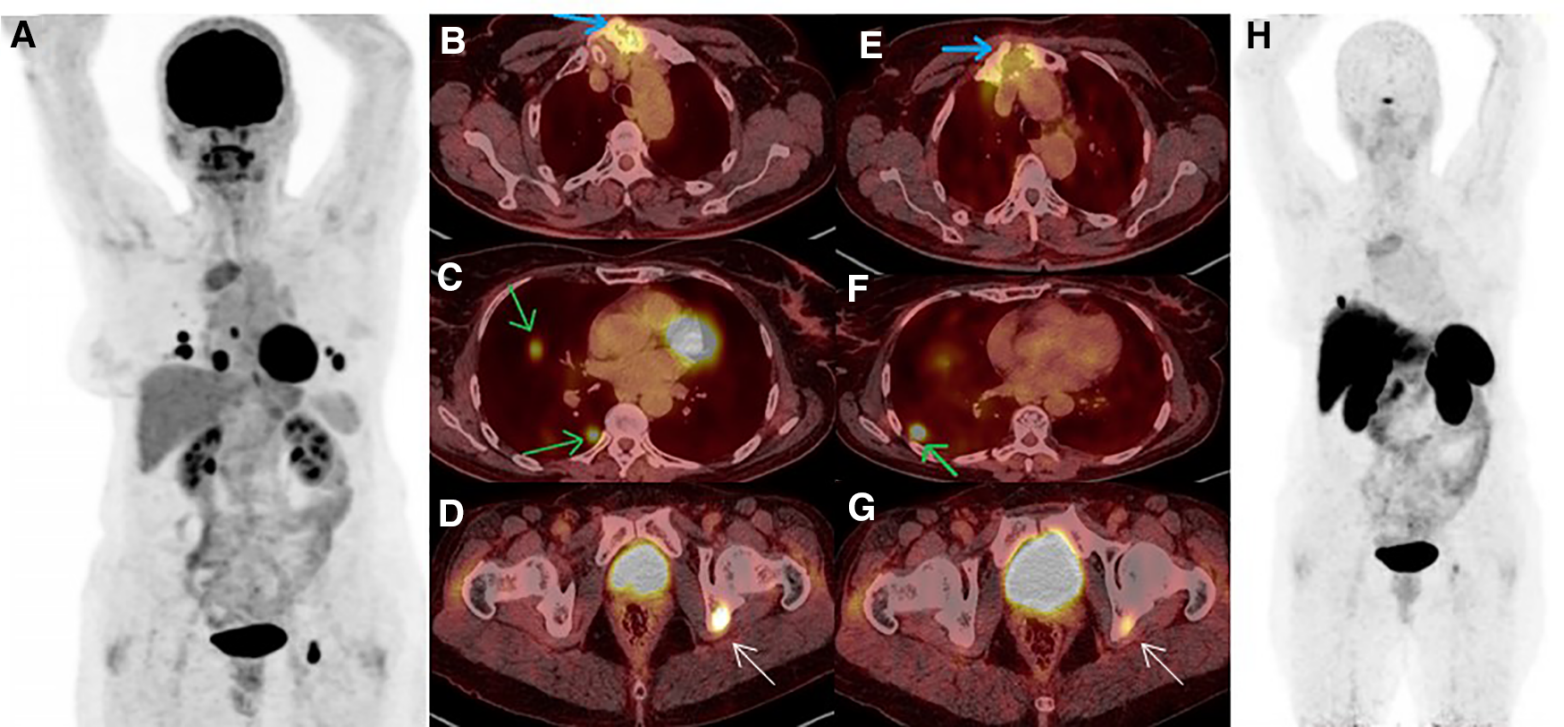
(**A**–**D**) ^18^F-FDG PET/CT MIP revealed increased tracer uptake in pulmonary nodules, sternal, and acetabular lesions (green, blue and white arrows, respectively). (**E**–**H**) A few days later, ^68^Ga-DOTA-TATE PET/CT confirmed somatostatin-receptor (SSTR) uptake in the above-mentioned areas, albeit most of the pulmonary nodules were non-SSTR-avid.

**Figure 3:**
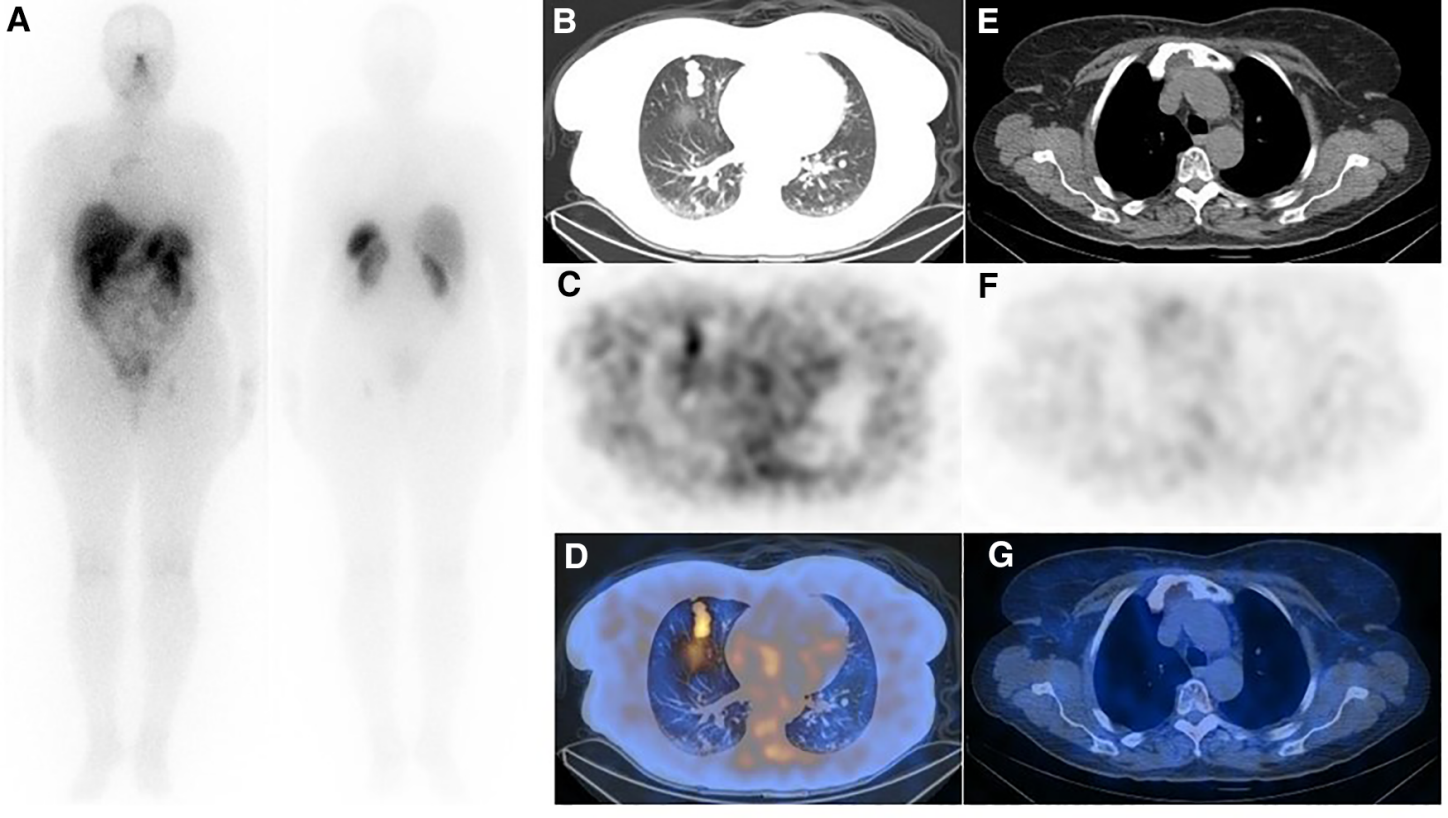
(**A**) Post-treatment whole-body imaging following administration of 200 mCi of ^177^Lu-DOTA-TATE (the second cycle is shown as an example). (**B**–**D**) Post-treatment SPECT/CT correlation showed moderate SSTR avidity in the lung metastases (**E**–**G**), while the uptake in the sternal lesion was unremarkable. Although interesting, the discordance of uptake in the pulmonary nodules and sternum between SSTR-targeted imaging (refer to figure 2) and therapy is confusing, and the underlying mechanism is unknown to us.

**Figure 4:**
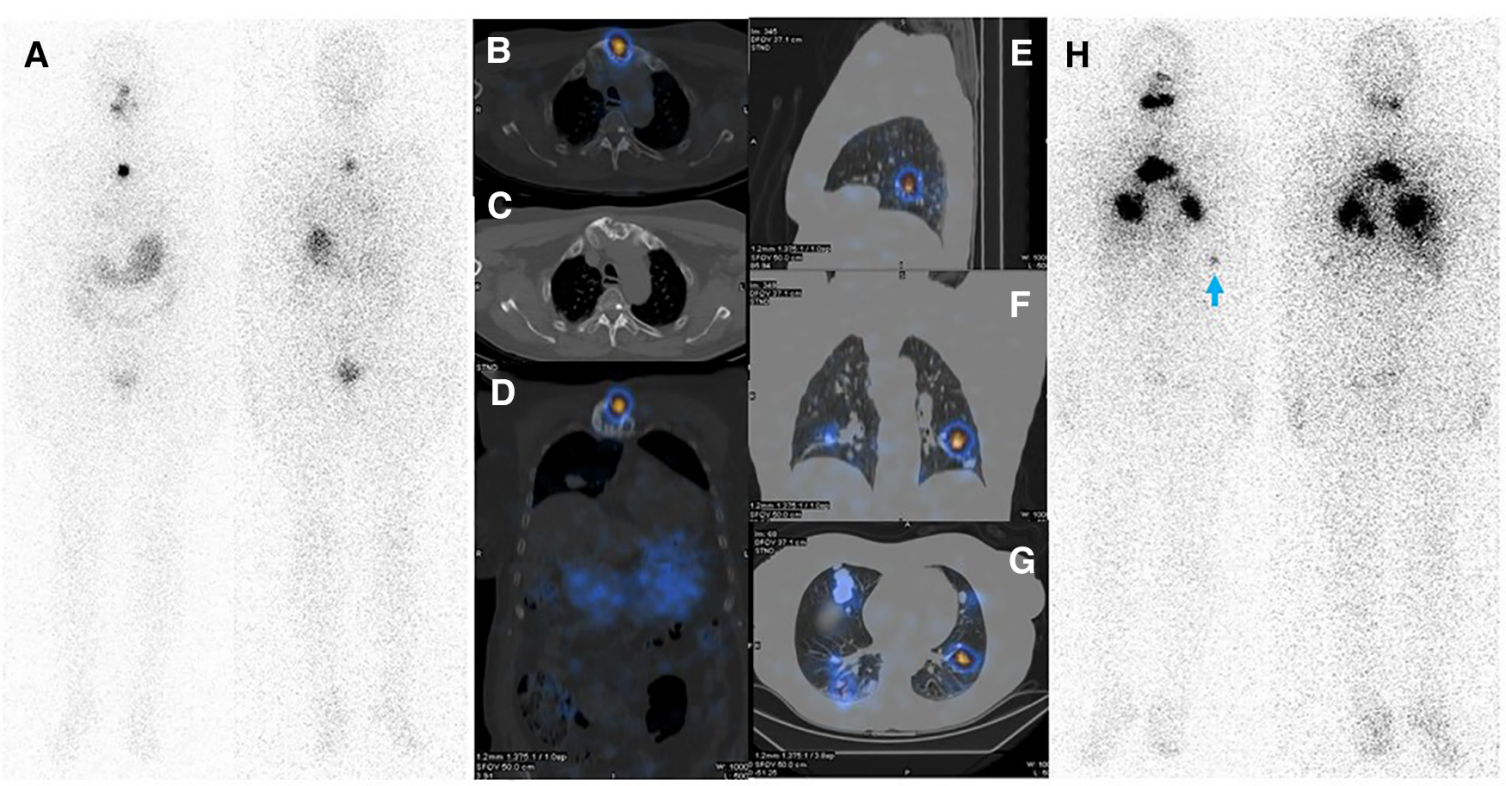
(**A**–**D**) Diagnostic WBIS following administration of 3 mCi of ^131^I showing regained sternal uptake. (**E**–**H**) Whole body WBIS after administration of 200 mCi of ^131^I depicted increased uptake in pulmonary metastases and a sternal lesion. Considering the appearance of new lesions as well as the increasing size of the main pulmonary nodules in the right middle lobe, disease progression is evident. Faint activity is also apparent in the left acetabulum (red arrowhead).

A 61-year-old woman with a history of follicular thyroid carcinoma (initial TNM: T_3a_N_0b_M_0_; restaging TNM: T_3a_N_1a_M_1_) was referred to our nuclear medicine department following a total thyroidectomy. She was under active follow-up for 11 years. The first stimulated serum Tg and anti-Tg antibody levels were 0.01 and 33 ng/ml, respectively. She received 30 mCi of I-131, and the post-ablation WBIS showed a post-surgical thyroid remnant (Figure 1A). During follow-up, she developed TENIS syndrome, and the stimulated Tg levels reached 351 ng/ml (Tg doubling time = 26 months) with negative diagnostic WBIS. After five years of follow-up, she complained of sternal pain, swelling, and elevated serum suppressed-Tg levels reaching >500 ng/ml. The lung CT correlation was suggestive of a lytic sternal lesion and bilateral pulmonary metastases (Figures 1B,C). Therefore, another dose of I-131 (200 mCi) was applied 13 months following a negative diagnostic WBIS, and the metastatic foci revealed no iodine avidity in the post-ablation scan (Figure 1D). Thus, the patient underwent external beam radiation therapy (EBRT) for the sternum. Subsequently, ^18^F-FDG PET/CT was done because of significantly rising serum-suppressed Tg (i.e., >30,000 ng/ml). Also, for evaluation of SSTR expression, ^68^Ga-DOTA-TATE PET/CT was performed. These two scans revealed multiple pulmonary nodules and a sternal lytic lesion with acceptable uptake of both radiotracers (Figure 2). After being discussed in the tumor board meeting, the patient was deemed eligible for PRRT, and four cycles of ^177^Lu-DOTA-TATE were administered (cumulative = 810 mCi). Post-treatment PRRT SPECT/CT depicted increased radiotracer uptake in the aforementioned areas (Figure 3). Following PRRT, clinical symptoms improved, and serum suppressed-Tg level was reduced to 1884 ng/ml. During PRRT, the patient experienced grade I thrombocytopenia according to the 5th version of the common terminology criteria for adverse events. On this account, the treating physician decided to withhold additional cycles requesting a diagnostic WBIS. Surprisingly, the WBIS showed significant iodine avidity in the sternal lesion (Figures 4A–D). The patient received a trial of 200 mCi ^131^I, and the post-treatment whole-body scan confirmed the regaining RAI uptake in all of the metastatic foci found in the SSTR PET/CT study (Figures 4E–H). The interval between this WBIS and the previous negative study was 70 months.

## Discussion

RAI therapy is the treatment of choice for differentiated thyroid carcinoma after total thyroidectomy, with about two-thirds of patients demonstrating appropriate radioiodine accumulation in metastatic sites (1). Nearly 30% of patients with progressive metastatic differentiated thyroid carcinoma develop a radioiodine-refractory disease (7). Treatment options are limited in these patients (8). In asymptomatic patients as well as in stable, or minimally progressive disease, monitoring during TSH suppression is recommended (9).

Interventions should be done only in symptomatic patients in order to decrease morbidity. Surgery, radiation therapy, PRRT or TKIs are alternative treatment options. However, because of the severe adverse effects of TKIs, they should be used cautiously (4). In patients with radioiodine-refractory thyroid carcinoma, PRRT is a therapeutic option with minimal toxicity and probably survival benefits (10, 11).

A comprehensive systematic review investigated the safety and efficacy of PRRT in treating progressive RAIR-DTC and metastatic medullary thyroid cancer. The results showed that PRRT could stabilize the disease with few side effects (5, 6). It needs over-expression of somatostatin receptor subtype II (10), which can be assessed by SSTR scintigraphy like ^68^Ga-DOTA-TATE PET/CT to determine the somatostatin receptor density in the metastases and recurrent tumor lesions as well as evaluating treatment response (12).

One may suggest that non-visualization of the metastatic foci following the second dose of RAI can be due stunning effect secondary to prior diagnostic WBIS. However, to the best of our knowledge, no study has pointed out the persistence of this phenomenon for more than several weeks to months (12–16). In the current case, the interval between diagnostic and therapeutic RAI was 13 months. Therefore, the stunning effect phenomenon is unlikely to be the plausible explanation for what we have observed here.

Despite doing a thorough literature search, we could not find any hypothetical mechanism for re-differentiation following PRRT. Indeed, data in this research domain is still limited, as fewer than 200 cases of RAIR-DTC have been treated with PRRT (5, 6). Whether regaining radioiodine uptake in the current case was serendipitously found during our observations or really happened because of PRRT-induced irradiation to the tumoral cells needs to be elucidated in future studies.

## Conclusion

We report a case of RAIR-DTC that showed re-differentiation and gained RAI uptake capacity after four cycles of PRRT. This hypothesis may be a ray of hope. It may propose PRRT as a possible re-differentiation strategy in patients with RAIR-DTC who have lost their RAI avidity if repeated and confirmed by future studies.

## Data Availability

The original contributions presented in the study are included in the article, further inquiries can be directed to the corresponding author.
